# Function and Mechanism of ZcucOBP14 in Regulating Olfactory Recognition and Insecticide Susceptibility in *Zeugodacus cucurbitae*

**DOI:** 10.3390/ijms27125158

**Published:** 2026-06-06

**Authors:** Jingjing Wang, Yang Yue, Chao Ma, Zhenya Tian, Yan Zhang, Hongsong Chen, Weihua Ma, Zhongshi Zhou

**Affiliations:** 1 State Key Laboratory for Biology of Plant Diseases and Insect Pests, Institute of Plant Protection, Chinese Academy of Agricultural Sciences, Beijing 100193, China; wjingjing1996@163.com; 2 National Nan fan Research Institute, Chinese Academy of Agricultural Sciences, Sanya 572019, China; 3 Guangxi Key Laboratory for Biology of Crop Diseases and Insect Pests, Institute of Plant Protection, Guangxi Academy of Agricultural Sciences, Nanning 530007, China; 4 Hubei Insect Resources Utilization and Sustainable Pest Management Key Laboratory, College of Plant Science and Technology, Huazhong Agricultural University, Wuhan 430070, China

**Keywords:** *Zeugodacus cucurbitae*, odorant-binding proteins, olfactory, molecular docking

## Abstract

The melon fly, *Zeugodacus cucurbitae* (Coquillett), is a globally significant agricultural pest causing substantial economic losses. Odorant-binding proteins (OBPs) are critical of the insect olfactory system, yet their specific physiological functions in *Z. cucurbitae* remain largely uncharacterized. In this study, we functionally characterized *ZcucOBP14* and investigated its putative involvement in host chemoreception and insecticide tolerance. Sequence alignment and phylogenetic analysis indicated that ZcucOBP14 belongs to the Minus-C OBP subfamily, and quantitative reverse transcription PCR (RT-qPCR) showed that it was predominantly expressed in both the head and abdomen. Fluorescence binding assays revealed that ZcucOBP14 exhibited broad binding affinity to 11 host plant volatiles, three sex pheromones, and two insecticides. Subsequent electroantennography (EAG) and behavioral bioassays identified isopulegol, 1-hexanol, linalool, and α-pinene as key ligands regulating the behavioral responses of *Z. cucurbitae*. RNA interference (RNAi)-mediated knockdown of *ZcucOBP14* significantly reduced EAG responses to key ligands, eliminated behavioral preference, and increased insecticide-induced mortality by 20%. Molecular docking further identified that Tyr71, Ile67, Trp50, Val107, Phe116 and Leu70 were critical residues involved in ligand interactions. Collectively, these findings highlight the indispensable role of ZcucOBP14 in olfactory perception and its contribution to insecticide tolerance, laying a solid theoretical foundation for the development of novel behavior-modifying agents, attractants, and optimized integrated pest management (IPM) strategies against this pest.

## 1. Introduction

Insects depend on a diverse array of chemosensory receptors within their olfactory system to detect and discriminate environmental odorants, thereby regulating a variety of essential behaviors, including host localization, foraging, oviposition, mate finding, and predator avoidance [1,2]. Odorant recognition is a complex process wherein environmental odorants bind to odorant-binding proteins (OBPs) or chemosensory proteins (CSPs), forming soluble OBP-odor complexes [3,4]. These complexes are then transported through hydrophilic sensillar lymph to odorant receptors (ORs) on olfactory receptor neurons (ORNs) [5,6], initiating signal transduction and ultimately modulating insect behavior [7].

Among the key molecules involved in this chemosensory cascade, insect chemosensory proteins comprise two major families: OBPs and CSPs. Both function as small, soluble carriers of hydrophobic odorants but exhibit distinct structural and functional characteristics [8]. OBPs typically contain 120–150 amino acid residues with conserved cysteines forming multiple disulfide bridges, stabilizing an α-helical hydrophobic binding cavity [9]. CSPs have a similar molecular weight but possess only four conserved cysteines forming two disulfide bonds, conferring greater conformational plasticity and stability under extreme conditions [10]. Functionally, OBPs are primarily involved in chemosensory and gustatory behaviors—including host location, mating, oviposition, and avoidance of toxic compounds—whereas CSPs participate not only in chemoreception but also in development, immunity, and plant–insect interactions. Regarding insecticide resistance, both families contribute through three main mechanisms: (1) direct binding and sequestration of insecticides, (2) synergistic regulation of detoxification enzymes, and (3) behavioral avoidance [11,12,13]. Crucially, as the first molecular filters in the chemosensory pathway, OBPs are indispensable for the initial detection and selective transport of odorants, laying the foundation for subsequent olfactory perception [14].

OBPs are a class of small, soluble extracellular proteins first identified in *Antheraea polyphemus* [15]. Structurally, OBPs are characterized by α-helical domains stabilized by three interlocked disulfide bridges formed by conserved cysteine residues [16]. A typical OBP possesses an N-terminal signal peptide and six conserved cysteines that form three disulfide bonds, which are critical for maintaining its structural integrity and ligand-binding capacity [17,18]. Based on the number and arrangement of conserved cysteine residues, OBPs are classified into five classes: Classical OBPs (six cysteines), Plus-C OBPs (eight cysteines), Minus-C OBPs (four cysteines), Dimer OBPs (characterized by dimerization and twelve cysteines), and Atypical OBPs (9–10 cysteines with an extended C-terminus) [19,20]. OBPs are multifunctional molecules essential for insect survival, primarily recognized for their role in olfactory perception, including the transport and discrimination of odorants such as host volatiles and pheromones [21,22,23], as well as the clearance of chemical signals to maintain sensory sensitivity [24,25]. Beyond olfaction, OBPs are involved in gustation, reproductive processes, and insecticide resistance through toxin sequestration [26,27], and even immune defense and visual processing [24,28]. This functional diversity underscores their remarkable functional diversity across different tissues and biological contexts.

The melon fly, *Zeugodacus cucurbitae* (Coquillett) (Diptera: Tephritidae), is a highly polyphagous and globally invasive pest capable of infesting over 100 host plant species, including economically important cucurbit crops such as cucumber, bitter gourd, sponge gourd, melon, and pumpkin [29]. Female adults oviposit into tender fruit tissues, and subsequent larval feeding causes fruit deformation, premature ripening, rotting, and abscission. In severely infested areas, yield losses can reach 30–100%, causing substantial economic losses estimated at hundreds of millions of dollars annually worldwide [30,31,32]. Currently, control strategies for *Z. cucurbitae* rely heavily on broad-spectrum insecticides; however, their intensive and long-term use has resulted in increased insecticide tolerance and concerning pesticide residues, compromising food safety and ecological health [31]. These challenges highlight the urgent need for alternative and eco-friendly control methods against this destructive pest. Female melon flies rely on olfaction to detect host plant volatiles for host localization [32,33]. Although the behavioral responses to various host volatiles have been extensively characterized [34,35], the molecular mechanisms underlying odorant detection and signal transduction remain largely unknown. Therefore, elucidating the functions of key chemosensory genes is essential for understanding the molecular basis of host-seeking behavior in this pest.

Although 35 OBPs have been identified in the antennae of *Z. cucurbitae* (NCBI BioProject PRJNA1019880) [36], their functional roles remain poorly characterized. *ZcucOBP14* was selected based on: (i) although membership in the Minus-C subfamily does not necessarily imply any specific function, members of this subfamily have been repeatedly linked to chemosensation and insecticide tolerance [37,38,39]; (ii) abundant abdominal expression suggesting broader physiological roles; and (iii) the broadest ligand-binding spectrum. In this study, the full-length cDNA of *ZcucOBP14* was cloned, and the recombinant protein was expressed and purified. Fluorescence competitive binding assays were employed to systematically evaluate the broadest ligand-binding spectrum of ZcucOBP14 to various ligands, including host plant volatiles, sex pheromones, and insecticides, as observed in our preliminary in vitro binding assays. Furthermore, RNA interference (RNAi), electroantennography (EAG), and behavioral assays were combined to assess the role of ZcucOBP14 in olfactory perception and behavioral regulation. Molecular docking was also performed to identify key amino acid residues involved in ligand-ZcucOBP14 interactions. Collectively, these findings are expected to provide a molecular basis for developing novel behavioral modifiers and support the design of eco-friendly control strategies against *Z. cucurbitae*.

## 2. Results

### 2.1. Sequence Analysis and Expression Profiles of ZcucOBP14

**Figure 1:**
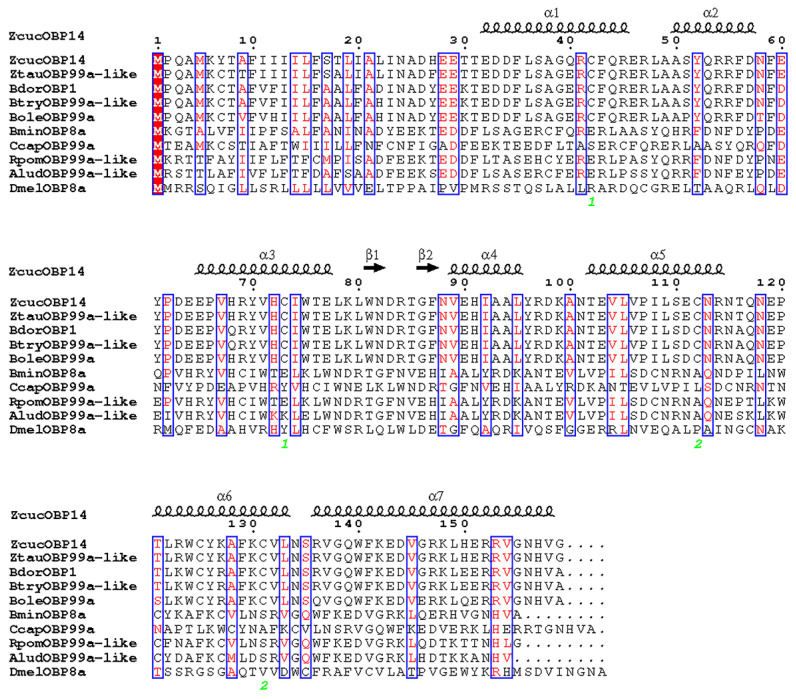
Alignment of the ZcucOBP14 with other dipteran OBPs. Sequence alignment was performed using the ClustalW algorithm with default parameters in MEGA 6.0 software. Species abbreviations: *Zeugodacus cucurbitae* (Zcuc); *Zeugodacus tau* (Ztau); *Bactrocera dorsalis* (Bdor); *Bactrocera tryoni* (Btry); *Bactrocera oleae* (Bole); *Bactrocera minax* (Bmin); *Ceratitis capitata* (Ccap); *Rhagoletis pomonella* (Rpom); *Anastrepha ludens* (Alud); *Drosophila melanogaster* (Dmel).

**Figure 2:**
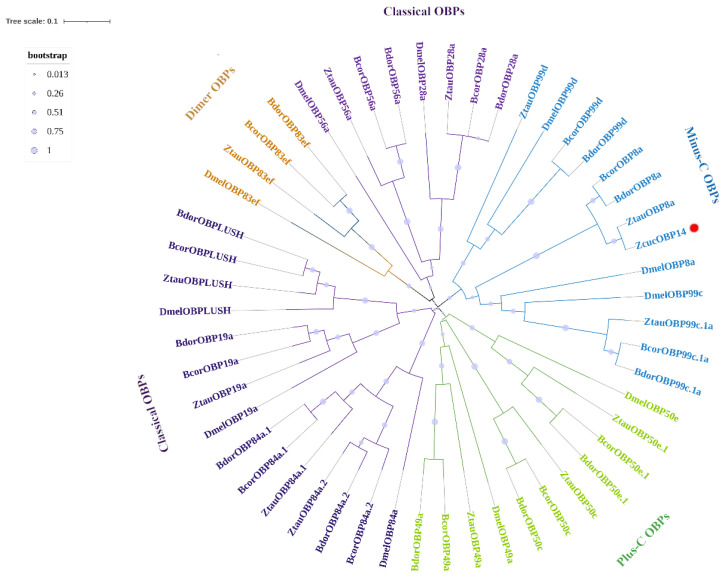
Phylogenetic analysis of ZcucOBP14 and other dipteran OBPs. The phylogenetic tree was constructed using the neighbor-joining (NJ) method with the p-distance model in MEGA v6.0 software. Node support was assessed using 1000 bootstrap replicates. The scale bar indicates a genetic distance of 0.1. Different OBP subfamilies are marked, including Classical OBPs, Plus-C OBPs, Minus-C OBPs, and Dimer OBPs.

**Figure 3:**
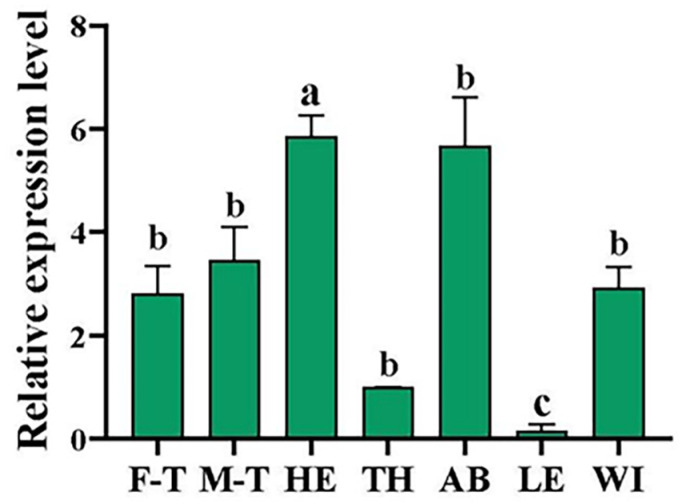
Expression profiles of *ZcucOBP14* in different tissues. Tissue abbreviations: M-T: male antennae, F-T: female antennae, HE: head, TH: thorax, AB: abdomen, LE: leg, and WI: wing. The relative expression level is indicated as the mean ± SE. Different letters indicate significant difference (ANOVA, LSD, *n* = 3, *p* < 0.05).

The *ZcucOBP14* gene was successfully cloned, with a full-length open reading frame of 667 bp that encoded 159 amino acids. The deduced ZcucOBP14 protein contains a signal peptide of 25 amino acids. The predicted molecular weight and isoelectric point were 21.5 kD and 6.1. Multiple sequence alignment results showed that the amino acid sequence of ZcucOBP14 contained four conserved cysteine residues and belonged to the Minus-C OBP family (Figure 1). The helical framework was stimulated using two highly conserved internal disulfide bridges comprising four cysteine residues: Cys42–Cys73 and Cys11–Cys131. Phylogenetic analysis divided OBPs into three branches, with ZcucOBP14 clustering with Minus-C OBPs from other dipterans (Figure 2). In addition, RT-qPCR results indicated that ZcucOBP14 was expressed in all tissues of *Z. cucurbitae*, with high expression levels in both the head and abdomen (Figure 3).

### 2.2. Binding Characteristics of Recombinant ZcucOBP14

**Figure 4:**
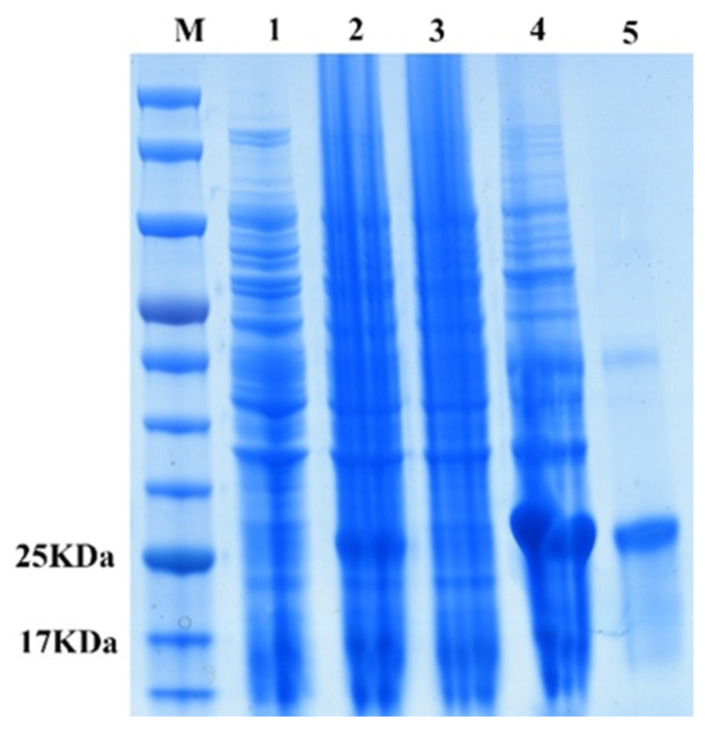
SDS-PAGE detection of recombinant protein ZcucOBP14 expression and purification. M: Protein molecular weight marker (10–180 kDa); Lane 1: non-induced protein; Lane 2: Induced protein; Lane 3: supernatant; Lane 4: Inclusion bodies; Lane 5: Purified protein.

**Figure 5:**
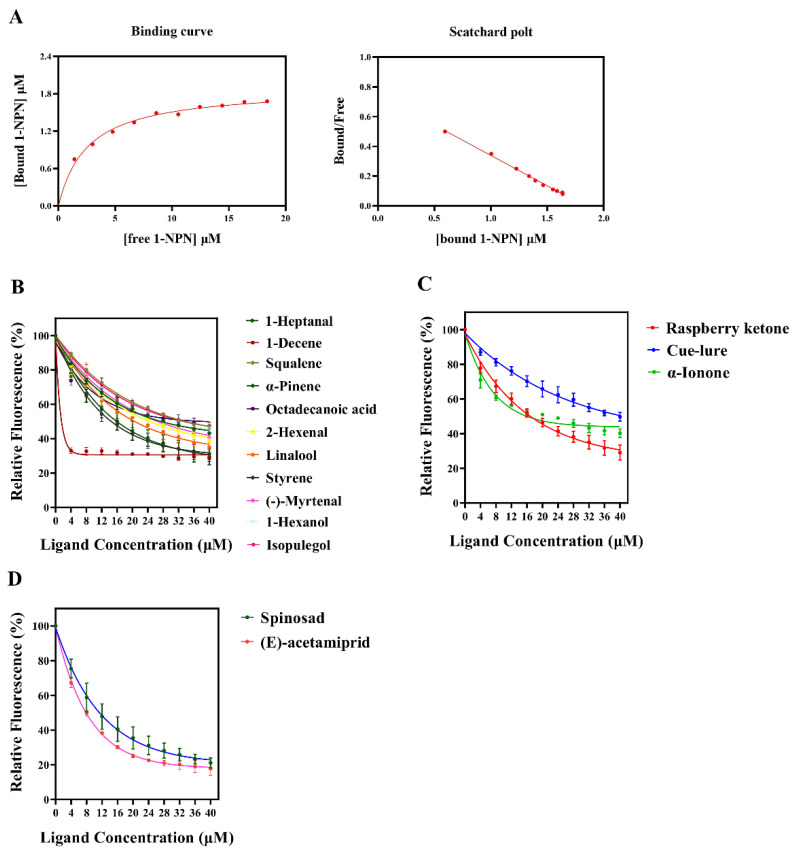
Ligand binding experiment of ZcucOBP14. (**A**) The binding curve of ZcucOBP14 to 1-NPN and Scatchard plots. (**B**) The binding curve of ZcucOBP14 and plant volatiles. (**C**) The binding curve of ZcucOBP14 and sex pheromones. (**D**) The binding curve of ZcucOBP14 and insecticides.

Recombinant ZcucOBP14 with a molecular weight of approximately 21.5 kDa was successfully expressed and purified (Figure 4). Fluorescence binding assay revealed that ZcucOBP14 could bind to 1-NPN with a binding constant (Kd) of 2.42 ± 0.59 μM (Figure 5A). Among all ligands tested, ZcucOBP14 has a broad binding capacity and binds to 11 plant volatiles, with the strongest binding affinity to 1-decene (Ki = 0.05 ± 0 μM) (Figure 5B). In addition, ZcucOBP14 could bind to three sex pheromones, including raspberry ketone (Ki = 16.8 ± 0.65 μM), cue-lure (Ki = 13.49 ± 0.52 μM), and α-ionone (Ki = 14.91 ± 0.58 μM) (Figure 5C). Notably, ZcucOBP14 displayed substantial binding affinity to two insecticides, spinosad (Ki = 8.65 ± 0.34 μM), and (E)-acetamiprid (Ki = 5.9 ± 0.23 μM) (Figure 5D). The IC50 and dissociation constant (Ki) values are listed in Table S4.

### 2.3. RNAi Assessment of ZcucOBP14

**Figure 6:**
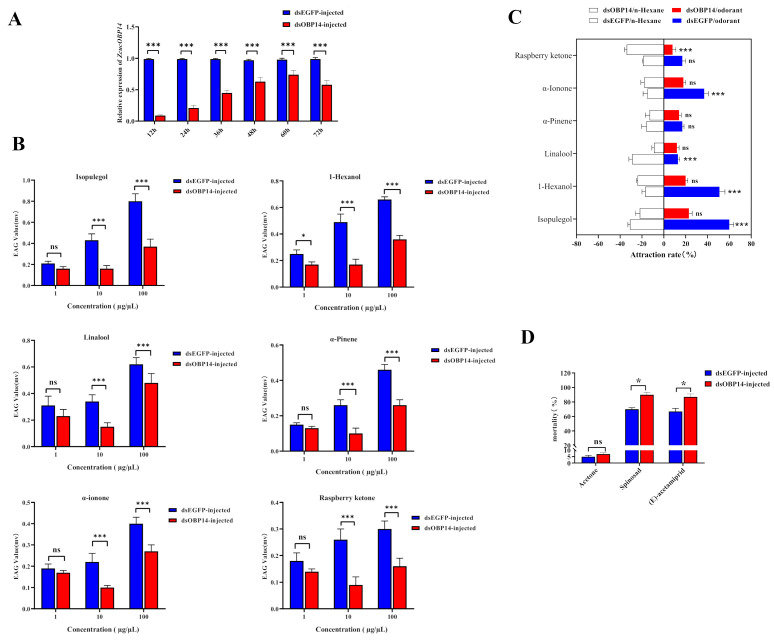
Functional verification of ZcucOBP14 via RNA interference. (**A**) The relative expression level of *ZcucOBP14* at different time points of RNAi. α-tubulin was selected as the internal reference gene. (**B**) EAG responses of dsOBP14-injected female adults to ligands at different doses. (**C**) Behavioral responses of dsOBP14-injected female adults to ligands. (**D**) Bioassay of dsOBP14-injected female adults to insecticides. Asterisks indicate significant differences (*t*-test, ns, no significant difference; * *p* < 0.05, *** *p* < 0.001).

To verify the RNAi effectiveness of *ZcucOBP14*, we examined the expression levels of *ZcucOBP14* at different time points after dsOBP14 injection. At 12 h post-injection of dsOBP14, the transcript level of *ZcucOBP14* was reduced by 90% compared with dsEGFP-injected controls, which was significantly greater than the other time points (Figure 6A). Therefore, melon flies 12 h post-injection were selected for subsequent assays.

Based on the results of the competitive fluorescence binding assay, EAG recordings further showed that these six ligands—isopulegol, 1-hexanol, linalool, α-pinene, α-ionone, and raspberry ketone—induced dose-dependent EAG responses in females (Figure 6B). Among them, α-ionone, 1-hexanol, linalool, and isopulegol were attractive to females (*p* < 0.05), whereas females showed no significant preference for raspberry ketone and α-pinene (Figure 6C). However, after silencing *ZcucOBP14*, females exhibited significantly reduced EAG responses to all six ligands at high concentrations (10 and 100 µg/µL) compared to the dsGFP-injected control group, while no significant differences were detected at the low concentration (1 µg/µL) (Figure 6B). Furthermore, following *ZcucOBP14* silencing, the behavioral preference for the four attractive ligands was lost, showing no significant difference from the control group (Figure 6C). In addition, dsOBP14-injected females exposed to spinosad and (E)-acetamiprid showed a significant 20% increase in mortality compared to controls (spinosad: *t* = 8.419, *p* = 0.001; (E)-acetamiprid: *t* = 9.733, *p* = 0.001) (Figure 6D). All raw data and statistical parameters were shown in Supplemental Material S1 and Tables S5–S7.

### 2.4. Homology Modeling and Molecular Docking

**Figure 7:**
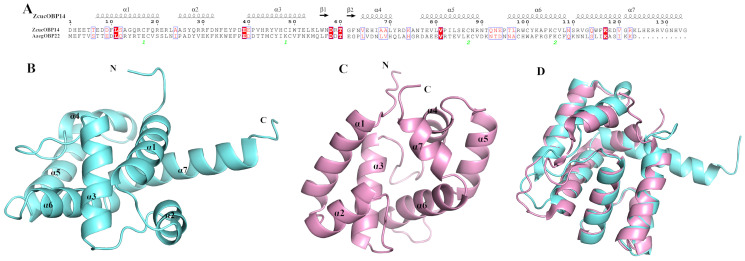
Three-dimensional structure model of ZcucOBP14. (**A**) Sequence alignment between ZcucOBP14 and AaegOBP22. (**B**) The 3D structures of ZcucOBP14. (**C**) The 3D structures of AaegOBP22. (**D**) Superimposed structure of ZcucOBP14 and AaegOBP22.

The three-dimensional structure of the mature ZcucOBP14 was predicted using AlphaFold3. The top-ranked ZcucOBP14 model exhibited a pTM score of 0.85 and was selected for subsequent quality assessment (Figure S2). Ramachandran plot analysis revealed that 116 residues (94.3%) were located in the favored region and 7 residues (5.7%) in the allowed region, whereas no residues fell within the disallowed region. The ERRAT overall quality factor of this model reached 100, further confirming its high structural quality (Figure S3). Taken together, these validations demonstrated that the AlphaFold3-predicted ZcucOBP14 model was reliable and structurally reasonable. A BLAST search against the PDB database showed that ZcucOBP14 shared the highest sequence identity (25%) with OBP22 from *Aedes aegypti* (PDB ID: 6NBN). Sequence alignment was further performed between ZcucOBP14 and AaegOBP22. Structural analysis demonstrated that the ZcucOBP14 model adopted the canonical architecture of Minus-C OBPs, consisting of four α-helices cross-linked by two conserved disulfide bonds (Figure 7).

**Figure 8:**
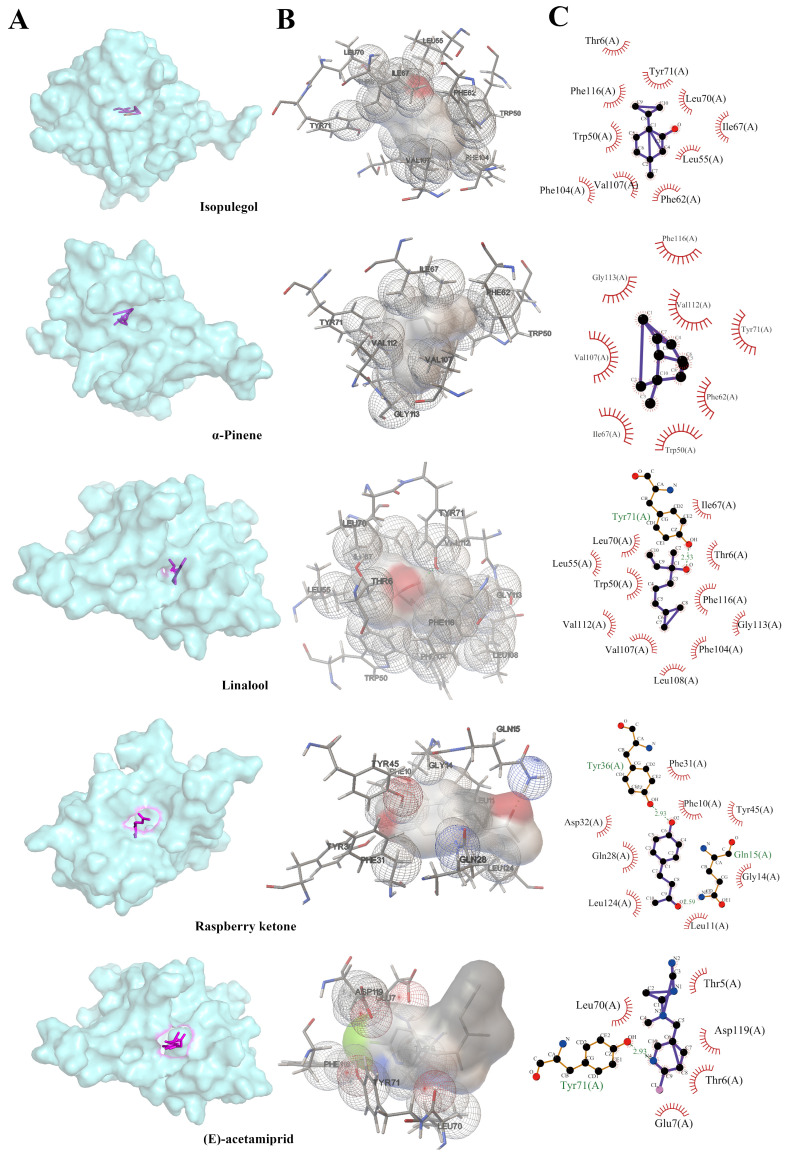
Interaction and binding models of ZcucOBP14 with representative ligands. (**A**) Overall binding posture of ZcucOBP14 and ligands. (**B**) 3D structure of ZcucOBP14 binding to ligands. (**C**) 2D structure of ZcucOBP14 binding to ligands.

The molecular docking results of ZcucOBP14 with the ligand showed that isopulegol exhibited the strongest binding affinity to ZcucOBP14 with a binding energy of −4.44 kcal/mol, followed sequentially by α-Pinene, linalool, raspberry ketone, and (E)-acetamiprid (Figure 8). All protein-ligand complexes were stabilized by combined hydrophobic interactions and hydrogen-bond networks. Specifically, α-pinene and isopulegol interacted exclusively through hydrophobic forces. By contrast, Linalool and (E)-acetamiprid relied on Tyr71 to form a hydrogen bond, while raspberry ketone relied on Tyr36 and Gln15 to build a hydrogen-bond network. These divergent interaction modes reflect the broad-spectrum molecular recognition capacity of ZcucOBP14 for structurally diverse ligands. The key conserved residues participating in ligand binding were Tyr71, Ile67, Trp50, Val107, Phe116 and Leu70. Detailed docking scores and interacting residues for each ligand are summarized in Table 1.

## 3. Discussion

OBPs are indispensable molecular mediators in insect olfactory recognition, serving as the first line of capturing and transporting external chemical signals [40,41]. Thus, elucidating the functional characteristics of OBPs in *Z. cucurbitae* is critical for developing targeted and environmentally sustainable control strategies. In this study, we identified and characterized the *ZcucOBP14* gene, which encodes a protein with a predicted molecular weight of 21.5 kDa and a 25-amino acid signal peptide. Sequence alignment confirmed that ZcucOBP14 belongs to the Minus-C OBP subfamily, characterized by four conserved cysteine residues. Phylogenetic analysis further revealed that ZcucOBP14 shared the highest homology with OBPs from other dipteran species, indicating strong evolutionary conservation and a shared ancestral origin.

Notably, *ZcucOBP14* was highly expressed in both the head and abdomen, reflecting distinct physiological functions. High expression in the head suggested its putative involvement in central olfactory signal processing, a characteristic common to head-specific Minus-C OBP [42]. Unlike antennally expressed OBPs that mediate peripheral chemical detection, head-localized OBPs often participate in signal integration and transduction within the central nervous system, highlighting ZcucOBP14’s potential role in higher-order olfactory processing beyond simple odorant transport [43,44]. In contrast, abundant expression in the abdomen implied a potential role in insecticide sequestration, metabolism, and tolerance, consistent with the dual functions of many detoxification-related OBPs [45,46].

To investigate the function of ZcucOBP14, we performed fluorescence binding assays using a diverse panel of 42 compounds, including 28 general plant volatiles [34,47], 11 dipteran sex pheromones [48], and five insecticides [49,50]. ZcucOBP14 exhibited broad ligand-binding capacity, interacting with 11 volatile compounds (with particularly high affinity for 1-decene, linalool, α-pinene, and styrene), three pheromones (raspberry ketone, cue-lure, and α-ionone), and two insecticides (spinosad and (E)-acetamiprid). Notably, its binding affinity for insecticides was significantly stronger, indicating that, beyond its role in general chemosensory recognition of environmental volatiles, it may also contribute to potential binding, sequestration and metabolic regulation of insecticides, highlighting its critical potential role in insecticide tolerance of *Z. cucurbitae* [51,52].

Complementary EAG and behavioral assays further corroborated the functional significance of these binding interactions. Key ligands such as α-pinene and α-ionone were attractive to *Z. cucurbitae*, while linalool was repellent, implicating these compounds in host-seeking and avoidance behaviors for survival and reproduction. RNAi-mediated knockdown of *ZcucOBP14* significantly reduced EAG responses to six ligands and abolished behavioral preference. Notably, silencing *ZcucOBP14* also induced a behavioral shift from neutral to repellent in response to raspberry ketone, underscoring its essential role in decoding chemical signals and translating them into adaptive behavioral outputs. Although the binding affinity of ZcucOBP14 for plant volatiles and pheromones was moderate, our RNAi, EAG, and behavioral assays firmly confirmed that this binding is essential for specific olfactory recognition, consistent with its head-specific expression pattern. This conclusion is further supported by three complementary lines of evidence: fluorescence binding assays demonstrating moderate-to-high affinity of ZcucOBP14 for candidate ligands, molecular docking validating specific binding modes, and EAG recordings showing that silencing ZcucOBP14 significantly reduces antennal responses to behaviorally relevant odorants. Collectively, these biochemical, structural, and physiological results reinforce the functional role of ZcucOBP14 in chemosensation.

Beyond its role in natural olfactory processes, ZcucOBP14 emerged as a key determinant in mediating insecticide susceptibility, a finding with significant implications for pest control [38]. The high abdominal expression of ZcucOBP14 was a typical feature of OBPs involved in insecticide resistance, where they help insects bind and sequester toxic substances to achieve detoxification and tolerance. In this study, ZcucOBP14 exhibited strong binding affinity for spinosad and (E)-acetamiprid, and its silencing increased the susceptibility of *Z. cucurbitae* to these insecticides. This supported a model in which ZcucOBP14 functions as a scavenger protein, intercepting insecticide molecules and preventing them from interacting with their target sites, thereby affecting insecticide susceptibility.

The observation that ZcucOBP14 binds to multiple plant volatiles and pheromones does not diminish its insecticide-related potential. Rather, the functional significance of its insecticide-binding capacity is demonstrated by two key findings: (i) significantly stronger binding affinity for insecticides compared to most volatiles, and (ii) increased insecticide susceptibility upon *ZcucOBP14* silencing. The broad binding profile is consistent with the role of many OBPs as general carriers of hydrophobic ligands, while the tissue-specific high expression in the abdomen further supports a specialized function in xenobiotic sequestration [53,54]. We therefore propose that ZcucOBP14 serves a potential dual role: olfactory recognition in the head and insecticide tolerance in the abdomen, with the latter being quantitatively more pronounced in terms of binding affinity and physiologically relevant, as demonstrated by RNAi bioassays.

We acknowledge that the expression dynamics of ZcucOBP14 following insecticide exposure were not examined in this study. Therefore, while our RNAi functional assay demonstrates that ZcucOBP14 affects insecticide susceptibility, whether this gene is transcriptionally induced under insecticide stress remains to be investigated. A systematic expression profiling of all OBP family members under insecticide exposure would provide a more comprehensive basis for candidate selection. In our initial screening, ZcucOBP14 was prioritized due to its significantly higher transcriptional abundance in the head and abdomen compared with most other OBPs, as well as its superior insecticide-binding affinity. Nevertheless, the possibility that other OBPs may be equally or more actively involved in insecticide tolerance cannot be excluded. Future studies should therefore perform time-course expression analyses of *Z. cucurbitae* OBPs following sublethal insecticide treatment. Such investigations will help identify additional OBPs responsive to insecticide stress. Moreover, combining RNAi or CRISPR-mediated knockdown of multiple high-response OBPs—individually and in combination—will help assess their relative contributions and potential functional redundancy. These efforts will not only clarify the regulatory mechanism of ZcucOBP14 under chemical stress but also address the broader question of how OBP family members collectively contribute to insecticide detoxification and resistance.

Molecular docking analysis further elucidated the structural basis of ZcucOBP14’s ligand-binding specificity, identifying hydrogen bonding and hydrophobic interactions as the primary forces stabilizing ligand-protein complexes. Key residues, including Tyr71, Ile67, Trp50, Val107, Phe116 and Leu70 were identified as critical determinants for complex stability. Hydrogen bonds, formed between carbonyl or nitrogen atoms of the ligands and specific receptor sites, enhance binding stability and specificity [22,55]. In contrast, hydrophobic interactions contribute to binding affinity through conformational flexibility, enabling diverse ligands to adopt multiple orientations within the binding pocket [56,57]. The co-existence of dominant hydrophobic interactions and flexible hydrogen-bonding sites endows ZcucOBP14 with both broad-spectrum odorant recognition and moderate binding specificity, which is conducive to the development of novel behavior-regulating agents for pest management. Meanwhile, the conserved hydrophobic core and variable polar binding sites highlight the structural adaptability of ZcucOBP14 toward different odor ligands. These structural insights not only advance our understanding of the olfactory recognition mechanism mediated by Minus-C OBPs in *Z. cucurbitae*, but also provide a rational structural basis for designing novel compounds that target the olfactory perception of this destructive pest.

## 4. Materials and Methods

### 4.1. Insects

The insects were originally obtained from rotting fruits in Nanning, Guangxi. After continuous laboratory domestication for more than 20 consecutive generations, the colony was maintained in rearing cages (30 cm × 30 cm × 30 cm) under controlled laboratory conditions: temperature 26 ± 1 °C, relative humidity 70 ± 10%, and a photoperiod of 14:10 (L:D) h. Adults were provided with an artificial diet consisting of yeast and sugar (1:2 ratio), along with water ad libitum.

### 4.2. RNA Extraction, cDNA Synthesis, and Gene Cloning

Total RNA was extracted from the antennae of *Z. cucurbitae* using TRIzol (Invitrogen, Carlsbad, CA, USA). First-strand cDNA was reverse-transcribed from 1 μg of total RNA using TransScript^®^ One-Step gDNA Removal and cDNA Synthesis SuperMix (Trans gen Biotech, Beijing, China).

Primers targeting the open reading frame of ZcucOBP14 (XP_011184701.1) were designed using Primer Premier 5.0 based on the antennal transcriptome data of *Z. cucurbitae* [36] (primer sequences were listed in Table S1). PCR amplification was carried out in a 25 µL reaction mixture containing 2 μL cDNA template (1 μg/μL), 1 μL each of forward and reverse primers (10 μM), 1 μL EasyTaq^®^ DNA Polymerase (5 U/μL; Trans Gen Biotech, Beijing, China), 0.5 μL dNTP (2.5 mM each), 2.5 μL 10× EasyTaq^®^ Buffer, and 17 μL RNase-free water. The PCR cycling conditions were as follows: 95 °C for 5 min; 35 cycles of 95 °C for 30 s, 55 °C for 30 s, 72 °C for 1 min; and a final extension at 72 °C for 10 min. The PCR products were resolved by 1% agarose gel electrophoresis, and target bands were purified using the Monarch^®^ gel extraction kit (NEB, Ipswich, MA, USA). The purified fragments were ligated into the pEASY-T3 vector and subsequently transformed into Trans-T1 chemoreceptor cells (Trans Gen Biotech, Beijing, China). Positive clones were screened on ampicillin-containing plates, and eight randomly selected clones were sent for sequencing. All kits were used according to the manufacturer’s protocol.

### 4.3. Sequence Analysis

The presence and location of the N-terminal signal peptides in ZcucOBP14 were predicted using SignalP 6.0 (https://services.healthtech.dtu.dk/services/SignalP-6.0/ (accessed on 5 April 2025)). The theoretical molecular weight and isoelectric point were calculated using the ExPASy Compute pI/Mw tool (https://web.expasy.org/compute_pi/ (accessed on 5 April 2025)). OBP protein sequences from other dipteran species were retrieved from the NCBI database (https://www.ncbi.nlm.nih.gov/ (accessed on 5 April 2025)), and multiple sequence alignment was performed using the ClustalW method in MEGA v6.0 (protein information is listed in Table S2). The phylogenetic trees were constructed using the neighbour-joining method with the p-distance model, and node support was assessed with 1000 bootstrap replicates. The final trees visualization and editing in FigTree v1.4.4 (protein information is listed in Table S3).

### 4.4. Tissue-Specific Expression Profiling of ZcucOBP14

Tissue samples (antennae from both sexes, heads, thoraxes, abdomens, legs, and wings) were collected from 15-day-old adult *Z. cucurbitae*, immediately frozen in liquid nitrogen, and stored at −80 °C. At least three biological replicates were analyzed per tissue type.

RT-qPCR was performed using SYBR Green-based RT-qPCR on an ABI 7500 Fast Real-Time PCR System (Applied Biosystems, Foster City, CA, USA). Primer sequences for ZcucOBP14 and the reference gene α-tubulin were listed in Table S1 [58]. Each 20 μL reaction contained 10 μL Hieff qPCR SYBR Green Master Mix (Trans gen Biotech, Beijing, China), 1 μL cDNA template, 0.4 μL of each primer (10 μM), and 8.2 μL RNase-free water. The PCR amplification program was: 95 °C, 5 min; 95 °C, 10 s; 60 °C, 30 s; 35 cycles, with default melt curve settings. Relative expression levels were calculated using the 2^−ΔΔCT^ method.

### 4.5. Expression and Purification of Recombinant ZcucOBP14

The coding sequence of ZcucOBP14 without the signal peptide was cloned into the pET-28a vector via *BamHI* and *NotI* restriction sites. The construct was sequence-verified (primers listed in Table S1) and transformed into BL21(DE3) competent cells (Yeasen Biotechnology, Shanghai, China). A single colony was inoculated into 5 mL LB medium containing 50 μg/mL kanamycin and cultured overnight at 37 °C with shaking at 220 rpm. The ZcucOBP14 protein expression was induced with 1 mM isopropyl β-D-thiogalactoside (IPTG) (Solarbio, Beijing, China) at 16 °C for 24 h. The bacterial cells were then harvested by centrifugation (10,000× *g*, 10 min, 25 °C), resuspended in 20 mL of 1× PBS, and lysed by sonication (200 W, ON 5 s/OFF 5 s, 10 min). An aliquot (1 mL) of the lysate was centrifuged (10,000× *g*, 2 min), 40 μL of supernatant was mixed with 10 μL of 5× SDS loading buffer (Lanbolide Trading, Beijing, China), and the pellet was resuspended in 40 μL ddH_2_O with 10 μL of 5× SDS loading buffer. All samples were boiled at 100 °C for 10 min and analyzed by SDS-PAGE.

Inclusion bodies were refolded using the redox method. They were resuspended in 30 mL of solution I (2 mL Triton-X-100, 6.057 g Tris, pH 6.8 with HCl, ddH_2_O to 1 L), and centrifuged (10,000× *g*, 20 min, 4 °C). The pellet was dissolved in 5 mL of 6 M guanidine hydrochloride, followed by the addition of 10 mL of solution II (0.31 g DTT, 4.85 g Tris, pH 6.0 with HCl, ddH_2_O to 0.2 L) and incubation at 25 °C for 1 h. Then, 4 mL of freshly prepared solution III (0.2 g NaOH, 0.24 g cystine in 10 mL ddH_2_O) was added, and the mixture was incubated at 25 °C for 10 min. The reaction solution was transferred to 10 volumes of solution IV (0.61 g cysteine, 12.1 g Tris, pH 8.0 with HCl, ddH_2_O to 1 L) and resuscitated at 90 rpm for 24 h at 25 °C. The solution was centrifuged (12,000× *g*, 20 min, 4 °C), filtered through a 0.22 μm PVDF membrane, and concentrated to about 50 mL using 10 kDa ultrafiltration tube. The molecular weight of the protein was verified using SDS-PAGE. All chemical reagents used in this refolding procedure were purchased from Solarbio, Beijing, China.

The recombinant His-tagged protein was purified by nickel affinity chromatography on the ÄKTA™ Avant 150 (General Electric, Boston, MA, USA) using a 0–100% linear imidazole gradient. The eluate fractions were concentrated with a 10 kDa ultrafiltration tube (Beaoyi Technology, Beijing, China) and the purity of the different fractions of protein was analyzed by SDS-PAGE. The purified protein was dialyzed at 4 °C against 50 mM Tris buffer (24.22 g Tris in 2 L ddH_2_O), which was refreshed after 2 h, followed by overnight dialysis. After ultrafiltration concentration, the protein concentration was determined using the BCA Protein Assay Kit (Solarbio, Beijing, China).

### 4.6. Fluorescence Binding Assay

The binding characteristics of ZcucOBP14 to ligands were investigated using an F-380 fluorescence spectrophotometer (Gangdong, Tianjin, China) with the following settings: excitation and emission slitat 10 nm, the excitation wavelength 337 nm, emission scan range from 360 to 500 nm, sensitivity 2 s, and scanning speed was 1200 nm/min. A panel of 44 ligands was evaluated, including 28 general plant volatiles from *Momordica charantia*, 11 sex pheromones of dipteran insects, and five insecticides. Detailed manufacturer information and purity of all these chemicals are listed in Table S8. All ligands and the fluorescent probe 1-NPN were dissolved in chromatographic methanol at 1 mM.

To test the binding of ZcucOBP14 to 1-NPN, the protein was diluted to 2 μM in 50 mM Tris-HCl (pH 7.4). Then, 2 mL of the protein solution was transferred into the cuvette, and 1-NPN was added in a gradient (0–20 μM, 2 μM intervals). After mixing and a 30 s equilibration, the maximum fluorescence intensity was recorded. For the competitive binding assay, 2 mL of 2 μM ZcucOBP14 solution was co-incubated with 2 μM 1-NPN for 30 s, followed by the addition of each competitor ligand at gradient concentrations (4 to 40 μM, 4 μM increments). Fluorescence intensities were measured in triplicate for each ligand concentration. The dissociation constant (Ki) was calculated using the Scatchard equation: Ki = [IC50]/(1 + [1-NPN]/K1-NPN), where IC50 is the ligand concentration reducing fluorescence by 50%, [1-NPN] is the free concentration of 1-NPN, and K1-NPN is the binding constant of the ZcucOBP14/1-NPN complex.

### 4.7. Gene Silencing by RNAi

Double-stranded RNA (dsRNA) templates were amplified by PCR using the T7 promoter primers and the PCR products were purified using the DNA extraction solution. The dsRNA was synthesized using the T7 RNAi Transcription Kit (NEB, Ipswich, MA, USA) according to the manufacturer’s instructions. The dsRNA was dissolved in RNase-free water and verified by concentration measurement and agarose gel electrophoresis. The purified dsRNA was diluted to 10 μg/μL and stored at −80 °C. After 15 days post-emergence, 200 nL of dsOBP14 was injected into the pronotum of the melon fly using Nanoliter 2000 microinjector (Drummond, Broomall, PA, USA). Flies injected with 200 nL of dsEGFP were used as the negative control. At 12, 24, 36, 48, 60 and 72 h post-injection, ten heads were collected and the interference efficiency of dsOBP14 at different time points was analyzed by RT-qPCR. Three biological replicates were performed for each time point.

### 4.8. Electroantennography and Behavioral Assays

At 12 h post-injection, the head was excised from adult *Z. cucurbitae*, the tip of the antenna (1 mm) was removed, and inserted into the recording electrode filled with 0.1 mol/L KCl, while the head was inserted into the reference electrode. Flies injected with dsEGFP served as controls. Each test compound was dissolved in n-hexane at 100 µg/µL, 10 µg/µL and 1 µg/µL, 10 µL of the solution was applied to filter paper (40 mm wide × 5 cm long), which was then placed in a 10 cm sample tube. Odor stimulus of 0.5 s was delivered to the antenna at a constant flow rate of 400 mL/min via an odor stimulation control device (CS-55, Syntech, Hilversum, The Netherlands). A 30 s interval was maintained between consecutive stimulations to allow antennal recovery. At least 15 different female flies were tested for each compound, and each antenna was used only once. EAG responses were normalized according to the formula: (compound response-control response), where the control response was the mean response to n-hexane before and after each stimulus. All data were tested for normal distribution, and any non-conforming data were excluded to ensure the reliability and accuracy of the results.

Trap assays were performed as described previously [59]. The olfactometer consisted of three cages (30 cm × 30 cm × 30 cm) connected by 5 cm holes, with the middle cage (R) for releasing flies, the T-cage for the odorant, and the C-cage for the n-hexane control (Figure S1). Each test compound was dissolved in n-hexane at 10 µg/µL, and 200 µL of the solution was applied to filter paper (2 cm wide × 2 cm long). At 12 h post-injection, melon flies were fasted for 6 h prior to the assay, and all assays were conducted in the dark to avoid visual interference. Sixty dsOBP14-injected female flies (15 days post-emergence) were introduced into the R cage at 10 am, and the number of melon flies entering T and C cages was counted at 6 pm. Flies injected with dsEGFP served as the control group. Each group was repeated at least five times, with the positions of the T and C cages swapped between replicates to eliminate contamination and directional bias. The attraction rate was calculated as follows: Attraction rate = (Number of flies in each trap/Total number of introduced flies) × 100%.

### 4.9. Insect Bioassay

The bioassays were conducted using the drug membrane method, and 750 mL plastic cups were used as film bottles [60]. Small holes were made in the cup lid for ventilation, and the center opening was plugged with a non-fat cotton ball soaked in 10% honey water for feeding. Based on unpublished laboratory data, the median lethal concentration (LC_50_) of Spinosad was 2 mg/L and (E)-acetamiprid was 20 mg/L, respectively. Stock solutions of 92% Spinosad and 98.1% (E)-acetamiprid powders were prepared with acetone at 5 g/L. For spinosad, the stock solution was diluted 1:2500 (*v*/*v*) with acetone to achieve a final concentration of 2 mg/L active ingredient. For (E)-acetamiprid, the stock solution was diluted 1:250 (*v*/*v*) with acetone to achieve a final concentration of 20 mg/L active ingredient. Then, 2.5 mL of the diluted insecticide solution was evenly applied to the inner wall of each cup, which was slowly rotated to form a uniform drug film. After 12 h of interference, thirty dsOBP14-injected female adults were placed into each cup, and dsEGFP-injected female adults served as controls. After 6 h of insecticide exposure, 10% honey water was supplemented for the melon flies to feed on, and mortality was recorded at 24 h. For the control group, an equal volume of pure acetone was evenly spread on the cup’s inner wall following the identical film-coating procedure to eliminate solvent interference. Bioassays exhibiting over 10% mortality in the control group were regarded as invalid. Five replicates were performed per treatment.

### 4.10. Homology Modeling and Molecular Docking

The three-dimensional (3D) structure of mature ZcucOBP14 was predicted using AlphaFold3 (https://alphafoldserver.com/ (accessed on 5 April 2025)). Homologous template sequences were retrieved from the Protein Data Bank (PDB, https://www.rcsb.org/ (accessed on 5 April 2025)), and sequence alignment and homology analysis were conducted using ESPript 3.0 (https://espript.ibcp.fr/ESPript/cgi-bin/ESPript.cgi (accessed on 5 April 2025)). The quality of the predicted structural model was comprehensively validated via the SAVES v6.0 web server (https://saves.mbi.ucla.edu/ (accessed on 5 April 2025)), including Ramachandran plot analysis and ERRAT evaluation, to verify the rationality and reliability of the protein structure.

The 3D structures of candidate odorant ligands were downloaded from the PubChem database (https://pubchem.ncbi.nlm.nih.gov/ (accessed on 5 April 2025)). Prior to docking, protein and ligand structures were preprocessed using AutoDock Tools 1.5.6, followed by molecular docking with AutoDock. Each ligand was docked in five independent runs to obtain averaged binding affinities for improved statistical reliability and reproducibility, with all grid parameters listed in Table S9. The lowest-energy conformation of each ligand was chosen as the optimal binding pose for interaction analysis, and ZcucOBP14–ligand binding modes were visualized accordingly. Structural visualization was carried out with PyMOL1.9.0 (Schrödinger, New York, NY, USA) and Discovery Studio Visualizer 2016 (Accelrys, San Diego, CA, USA). Key binding sites were analyzed using LigPlot+ v.2.3 software (EMBL-EBI, Hinxton, UK).

### 4.11. Data Analysis

All statistical analyses and figures were generated using GraphPad Prism 8. RT-qPCR data were analyzed using one-way ANOVA with Tukey’s multiple comparison tests. EAG, behavioral and bioassay experiments were analyzed using Student’s *t*-test. *p* < 0.05 was considered significantly different. A significance threshold of *p* < 0.05 was used throughout. All figures and tables now explicitly indicate significant differences with asterisks (* *p* < 0.05, ** *p* < 0.01, *** *p* < 0.001) and error bars are defined as mean ± SEM.

## 5. Conclusions

ZcucOBP14 functions as a pivotal chemosensory protein in *Z. cucurbitae*, with critical roles in olfactory recognition, behavioral regulation, and insecticide susceptibility. Functional characterization revealed that ZcucOBP14 binds to diverse ligands, and its silencing impairs electrophysiological responses and behavioral preferences to key ligands while increasing insecticide susceptibility. Structural analysis further identifies Tyr71, Ile67, Trp50, Val107, Phe116 and Leu70 as essential residues mediating these specific interactions. Collectively, these findings establish ZcucOBP14 as a promising target for behavior-modifying agents and insecticide synergists, offering an environmentally sustainable alternative to conventional pest control strategies. However, further investigation is needed to determine whether ZcucOBP14 expression is induced upon insecticide exposure, which would clarify its precise role in insecticide tolerance. Future validation of key residues in vivo and field evaluation of ZcucOBP14-targeted compounds will facilitate the integrated management of *Z. cucurbitae*.

## Figures and Tables

**Table 1:** Binding energy and key amino acid residues of ZcucOBP14-ligand interactions.

Ligands	Average Binding Energy (Kcal/mol)	Key Amino Acid Residues	
		Polar	Nonpolar
Isopulegol	−4.44	Thr6, Tyr71	Phe116, Leu70, Trp50, Ile67, Leu55, Phe104, Val107, Phe62
α-Pinene	−4.28	Tyr71, Gly113	Phe116, Val112, Phe62, Trp50, Ile67, Val107
Linalool	−3.58	Thr6, Tyr71, Gly113	Ile67, Phe116, Phe104, Leu108, Val107, Val112, Trp50, Leu55, Leu70
Raspberry ketone	−3.32	Tyr36, Tyr45, Glu15, Gly14, Gln28, Asp32	Phe31, Phe10, Leu11, Leu124
(E)-acetamiprid	−3.04	Thr5, Thr6, Tyr71, Glu7, Asp119	Leu70

## Data Availability

The original data presented in this study are openly available within the article and the Supplementary Materials.

## Supplementary Materials

The following supporting information can be downloaded at: https://www.mdpi.com/article/10.3390/ijms27125158/s1.
