# Supplementary material for: Function and Mechanism of ZcucOBP14 in Regulating Olfactory Recognition and Insecticide Susceptibility in Zeugodacus cucurbitae
Source: Int J Mol Sci. 2026 Jun 6;27(12):5158. doi: 10.3390/ijms27125158 (PMC13299693; doi:10.3390/ijms27125158)
Supplement: Supplementary file 1 [file ijms-27-05158-s001.zip › Supplementary Material 2-5.31.pdf]

Supplementary Materials

Table S1. The primer sequences of *ZcucOBP14* for cloning, RT-qPCR and dsRNA

Primer Name	Primer Sequences (5'-3')	Purposes
ZcucOBP14-F	ATGCACGCCTTAAACTCTC	For cloning
ZcucOBP14-R	TTATATTTTATGATAAATCACCT	
ZcucOBP14-F-q	CCTCCTATCAGCGTCGTTTC	For RT-qPCR
ZcucOBP14-R-q	CGGTTCATTTTGTGTATTCC	
α-Tubulin-F	CGCATTCATGGTTGATAACG	
α-Tubulin -R	GGGCACCAAGTTAGTCTGGA	
ZcucOBP14-eF	GGATCCGATCACGAGGAGACAACGG	For prokaryotic expression
ZcucOBP14-eR	GCGGCCGCTCAACCAACATGATTTCCCACA	
dsZcucOBP14-F	TAATACGACTCACTATAGGGATGCCACAAGCAATGAAG	For dsRNA synthesis
dsZcucOBP14-R	TAATACGACTCACTATAGGGTGAACCACTGACCCACCC	
dsEGFP-F	TAATACGACTCACTATAGGGAACTGGCGGGAACGGTAACTA	
dsEGFP-R	TAATACGACTCACTATAGGGCCTCCTGCATCTCAGGTGGTA	

Table S2. Protein sequence supplementary information

Species	Protein name	Accession number
Zeugodacus tau	ZtauOBP99a-like	ALS40426.1
Bactrocera dorsalis	BdorOBP1	AGS08183.1
Bactrocera tryoni	BtryOBP99a-like	XP_039948026.1
Bactrocera oleae	BoleOBP99a	XP_014090680.1
Bactrocera minax	BminOBP8a	AYN70626.1
Ceratitis capitata	CcapOBP99a	XP_004521185.1
Rhagoletis pomonella	RpomOBP99a-like	XP_036324633.1
Anastrepha ludens	AludOBP99a-like	XP_053946861.1
Drosophila melanogaster	DmelOBP8a	ACY92870.1

Table S3. Phylogenetic tree protein sequence supplementary information

Species	Protein name	Accession number	Group
	ZtauOBPLUSH	QKN21533.1	Classic
	ZtauOBP19a	QKN21535.1	Classic
	ZtauOBP28a	QKN21542.1	Classic
	ZtauOBP56a	QKN21547.1	Classic
	ZtauOBP84a.1	QKN21560.1	Classic
	ZtauOBP84a.2	QKN21561.1	Classic
Zeugodacus tau	ZtauOBP8a	QKN21534.1	Minus-c
	ZtauOBP99c.1a	QKN21563.1	Minus-c
	ZtauOBP99d	QKN21565.1	Minus-c
	ZtauOBP49a	QKN21544.1	Plus-C
	ZtauOBP50c	QKN21545.1	Plus-C
	ZtauOBP50e.1	QKN21546.1	Plus-C
	ZtauOBP83ef	QKN21558.1	Dimer
Bactrocera dorsalis	BdorOBPLUSH	QKN21308.1	Classic
	BdorOBP19a	QKN21310.1	Classic
	BdorOBP28a	QKN21317.1	Classic
	BdorOBP56a	QKN21323.1	Classic
	BdorOBP84a.1	QKN21335.1	Classic
	BdorOBP84a.2	QKN21336.1	Classic

	BdorOBP8a	QKN21309.1	Minus-c
	BdorOBP99c.1a	QKN21338.1	Minus-c
	BdorOBP99d	QKN21342.1	Minus-c
	BdorOBP49a	QKN21319.1	Plus-C
	BdorOBP50c	QKN21320.1	Plus-C
	BdorOBP50e.1	QKN21321.1	Plus-C
	BdorOBP83ef	QKN21333.1	Dimer
Bactrocera correcta	BcorOBPLUSH	QKN21085.1	Classic
	BcorOBP19a	QKN21087.1	Classic
	BcorOBP28a	QKN21094.1	Classic
	BcorOBP56a	QKN21100.1	Classic
	BcorOBP84a.1	QKN21112.1	Classic
	BcorOBP84a.2	QKN21113.1	Classic
	BcorOBP8a	QKN21086.1	Minus-c
	BcorOBP99c.1a	QKN21115.1	Minus-c
	BcorOBP99d	QKN21118.1	Minus-c
	BcorOBP49a	QKN21096.1	Plus-C
	BcorOBP50c	QKN21097.1	Plus-C
	BcorOBP50e.1	QKN21098.1	Plus-C
	BcorOBP83ef	QKN21110.1	Dimer
Drosophila melanogaster	DmelOBPLUSH	NP_524162.1	Classic
	DmelOBP19a	NP_728338.2	Classic

	DmelOBP28a	NP_523505.1	Classic
	DmelOBP56a	NP_611442.1	Classic
	DmelOBP84a	NP_001097700.2	Classic
	DmelOBP8a	NP_727322.1	Minus-c
	DmelOBP99c	NP_651711.1	Minus-c
	DmelOBP99d	NP_651712.1	Minus-c
	DmelOBP49a	NP_610812.1	Plus-C
	DmelOBP50c	NP_725387.3	Plus-C
	DmelOBP50e	NP_610959.2	Plus-C
	DmelOBP83ef	NP_731042.1	Dimer

Table S4. Competitive binding dissociation constant Ki of ZcucOBP14 with ligands

Compound types	Name of Compound	IC50 (μM)	Ki (μM)	CAS
Aldehydes	Hexanal	-	-	66-25-1
	(-)-Myrtenal	26.1±2.29	19.59±0.76	18486-69-6
	2-Hexenal	27.9±3.5	20.94±0.81	6728-26-3
	1-Heptanal	28.86±0.95	21.66±0.84	111-71-7
Alcohols	Isopulegol	-	-	23283-97-8
	Linalool	18.16±3.28	13.63±0.53	78-70-6
	(E)-4-hexen-1-ol	-	-	928-92-7
	(-)-myrtenol	-	-	19894-97-4
	1-Hexanol	-	-	111-27-3
Alkanes	Tetradecane	-	-	629-59-4
	Pentadecane	-	-	629-62-9
	Hexadecane	-	-	544-76-3
	Heptadecane	-	-	629-78-7
	Heneicosane	-	-	629-94-7
Esters	Methyl myristate	-	-	124-10-7
	Methyl palmitate	-	-	112-39-0
	Ethyl palmitate	-	-	628-97-7
	Methyl stearate	-	-	112-61-8
	Ethyl stearate	-	-	111-61-5

	Methyl isovalerate	-	-	556-24-1
	Squalene	34.55±0.07	25.93±1.00	111-02-4
	1-Decene	0.11±0.05	0.05±0	872-05-9
	1-Tetradecene	-	-	1120-36-1
Alkenes	*α*-Pinene	16.45±3.66	12.34±0.48	80-56-8
	1-Octadecene	-	-	112-88-9
	Styrene	13.99±2.08	10.5±0.41	100-42-5
	n-Hexadecanoic acid	-	-	57-10-3
	Octadecanoic acid	47.97±7.4	36±1.39	57-11-4
Acids	Myristic acid	-	-	544-63-8
	Eicosanoic acid	-	-	506-30-9
	(E)-acetamiprid	7.86±0.5	5.9±0.23	155569-91-8
	Spinosad	11.53±2.88	8.65±0.34	168316-95-8
Insecticides	Imidacloprid	-	-	138261-41-3
	Cyhalothrin	-	-	68359-37-5
	Methyl Eugenol	-	-	93-15-2
	2-allyl-4,5-dimethoxyphenol	-	-	59893-87-7
Sex pheromone analogue	(E)-coniferyl alcohol	-	-	458-35-5
	Eugenol	-	-	97-53-0
	Isoeugenol	-	-	97-54-1
	Methyl-isoeugenol	-	-	93-16-3

Raspberry ketone	22.39±3.16	16.8±0.65	5471-51-2
cue-lure	17.98±2.32	13.49±0.52	3572-06-3
Anisyl acetone	-	-	104-20-1
Zingerone	-	-	122-48-5
α-ionone	19.87±0.79	14.91±0.58	127-41-3
1-nonanol	-	-	143-08-8
1,3-nonanediol	-	-	23433-07-0
Nonyl Acetate	-	-	143-13-5

Table S5. Analysis of data of RNAi

Time (h)	*t*	*P*
12	91.936	<0.0001
24	39.456	<0.0001
36	23.042	<0.0001
48	10.475	<0.0001
60	9.557	<0.0001
72	12.545	<0.0001

Table S6. Analysis of data of EAG responses

Ligand	100 μg/μL		10 μg/μL		1μg/μL	
	t	*P*	*t*	*P*	*t*	*P*
Isopulegol	11.94	<0.0001	12.49	<0.0001	1.64	0.124
1-Hexanol	7.98	<0.0001	12.02	<0.0001	2.46	0.027
Linalool	4.62	<0.0001	9.15	<0.0001	2.57	0.022
α-Pinene	5.09	<0.0001	10.70	<0.0001	1.038	0.317
α-Ionone	3.84	0.002	7.76	<0.0001	0.869	0.4
Raspberry ketone	3.79	0.002	9.84	<0.0001	1.69	0.114

Table S7. Analysis of data of behavioral responses

Ligand	dsEGFP		dsOBP14	
	t	*P*	*t*	*P*
Isopulegol	9.997	<0.0001	0.496	0.633
1-Hexanol	9.816	<0.0001	1.088	0.308
Linalool	6.873	<0.0001	2.028	0.077
α-Pinene	0.862	0.414	0.772	0.463
α-Ionone	8.069	<0.0001	0.187	0.856
Raspberry ketone	0.792	0.451	8.272	<0.0001

Table S8. Manufacturer information and purity of all chemical reagents

Compound types	Name of Compound	Manufacturer	Purity (%)	CAS
	Hexanal	Sigma	≥95	66-25-1
	(-)-Myrtenal	Macklin	98	18486-69-6
Aldehydes	2-Hexenal	Sigma	98	6728-26-3
	1-Heptanal	Sigma	≥95	111-71-7
	Isopulegol	Macklin	99	23283-97-8
	Linalool	Yuanye	≥98	78-70-6
	(E)-4-hexen-1-ol	Macklin	97	928-92-7
Alcohols	(-)-myrtenol	Macklin	95	19894-97-4
	1-Hexanol	Sigma	98	111-27-3
	Tetradecane	Yuanye	≥99	629-59-4
	Pentadecane	Yuanye	≥99.5	629-62-9
	Hexadecane	Yuanye	≥99.5	544-76-3
Alkanes	Heptadecane	Yuanye	≥99.5	629-78-7
	Heneicosane	Yuanye	≥99	629-94-7
	Methyl myristate	Macklin	≥99.5	124-10-7
	Methyl palmitate	Sigma	≥99	112-39-0
Esters	Ethyl palmitate	Sigma	≥99	628-97-7
	Methyl stearate	Macklin	≥99.5	112-61-8
	Ethyl stearate	Macklin	99	111-61-5

	Methyl isovalerate	Sigma	≥99	556-24-1
	Squalene	Sigma	≥98	111-02-4
	1-Decene	Sigma	≥99	872-05-9
	1-Tetradecene	Sigma	≥97	1120-36-1
Alkenes	α-Pinene	Sigma	≥98	80-56-8
	1-Octadecene	Sigma	≥99	112-88-9
	Styrene	Sigma	≥98	100-42-5
	n-Hexadecanoic acid	Sigma	≥99	57-10-3
	Octadecanoic acid	Sigma	≥97	57-11-4
Acids	Myristic acid	Sigma	≥98	544-63-8
	Eicosanoic acid	Sigma	≥99	506-30-9
	(E)-acetamiprid	Yuanye	≥95	155569-91-8
	Spinosad	Yuanye	≥90	168316-95-8
Insecticides	Imidacloprid	Yuanye	≥95	138261-41-3
	Cyhalothrin	Sigma	≥95	68359-37-5
	Methyl Eugenol	Sigma	≥98	93-15-2
	2-allyl-4,5-dimethoxyphenol	Yuanye	95	59893-87-7
Sex pheromone analogue	(E)-coniferyl alcohol	Sigma	≥97.5	458-35-5
	Eugenol	Sigma	≥98	97-53-0
	Isoeugenol	Sigma	99	97-54-1
	Methyl-isoeugenol	Sigma	≥98	93-16-3

	Raspberry ketone	Sigma	99	5471-51-2
	cue-lure	Sigma	≥96	3572-06-3
	Anisyl acetone	Sigma	≥98	104-20-1
	Zingerone	Sigma	≥98	122-48-5
	α-ionone	Sigma	≥96	127-41-3
	1-nonanol	Macklinasx	≥99.5%	143-08-8
	1,3-nonanediol	MCE	95	23433-07-0
	Nonyl Acetate	Sigma	≥97	143-13-5

Table S9. Docking grid parameters for ZcucOBP4 and ligands

Ligand	Binding energy (Kcal/mol)	Grid dimensions			Grid spacing (Å)	Center Grid Box		
		x	y	z		x	y	z
Raspberry ketone-1	-3.87	110	90	80	0.492	4.08	-1.234	0.383
Raspberry ketone-2	-3.79	110	80	72	0.581	4.08	-1.234	0.383
Raspberry ketone-3	-2.91	114	86	72	0.581	0.23	-1.234	0.383
Raspberry ketone-4	-3.06	110	90	80	0.492	4.08	-1.234	0.383
Raspberry ketone-5	-2.96	126	126	126	0.436	3.856	-6.665	7.736
Isopulegol-1	-3.76	126	126	126	0.431	2.947	3.903	8.578
Isopulegol-2	-4.24	126	88	84	0.469	2.623	-1.234	0.383
Isopulegol-3	-4.94	126	116	90	0.431	2.623	-1.234	0.383
Isopulegol-4	-4.92	126	106	90	0.431	4.238	-1.234	0.383
Isopulegol-5	-4.34	126	106	90	0.431	4.238	-1.234	0.383
α-Pinene-1	-3.92	126	126	126	0.464	2.882	-1.234	9.615
α-Pinene-2	-4.49	126	106	90	0.431	4.238	-1.234	0.383
α-Pinene-3	-4.45	126	102	96	0.431	4.238	-0.535	0.383
α-Pinene-4	-4.22	126	100	90	0.447	5.184	-0.535	0.383
α-Pinene-5	-4.33	126	100	90	0.447	5.184	-0.954	0.383
Linalool-1	-3.17	126	126	126	0.453	5.778	0.179	-4.339
Linalool-2	-3.33	126	90	88	0.464	5.431	-1.141	-0.699
Linalool-3	-3.66	126	100	90	0.436	4.18	-0.34	0.383
Linalool-4	-3.9	126	104	88	0.436	4.54	-1.141	1.061
Linalool-5	-3.82	126	100	90	0.453	3.649	1.364	0.383
(E)-acetamiprid-1	-3.19	126	126	126	0.469	1.78	-7.892	5.933
(E)-acetamiprid-2	-2.97	118	108	88	0.458	4.229	-1.141	-0.699
(E)-acetamiprid-3	-2.98	118	108	104	0.458	4.564	-1.141	3.685
(E)-acetamiprid-4	-2.68	126	100	100	0.453	3.293	1.204	3.685
(E)-acetamiprid-5	-3.4	126	100	100	0.453	3.293	1.204	3.685
1-Hexenol-1	-2.34	126	126	126	0.375	0.23	-1.234	0.383
1-Hexenol-2	-2.41	110	90	92	0.519	5.623	-1.234	0.383
1-Hexenol-3	-2.44	110	90	90	0.519	5.15	-1.234	0.383
1-Hexenol-4	-2.39	110	90	90	0.508	3.929	-1.234	0.383
1-Hexenol-5	-2.45	115	100	90	0.481	3.929	-1.234	0.383
spinosad -1	2.05	120	100	90	0.481	5.296	-1.234	0.383
spinosad -2	1.32	126	120	90	0.453	5.036	-4.641	0.383
spinosad -3	1.93	120	100	100	0.453	5.036	0.551	-2.891
spinosad -4	0.66	126	100	100	0.453	5.255	-0.484	-1.727
spinosad -5	1.04	120	120	90	0.453	3.862	3.942	-0.44
α-ionone-1	5.56	126	126	126	0.436	3.812	-1.234	0.383
α-ionone-2	5.34	120	100	80	0.475	3.812	-1.234	0.383
α-ionone-3	4.98	120	120	90	0.475	3.812	-1.162	0.383
α-ionone-4	5.6	110	100	80	0.508	4.28	-3.399	1.302

α-ionone-5	4.46	120	100	80	0.464	4.958	-1.234	0.383

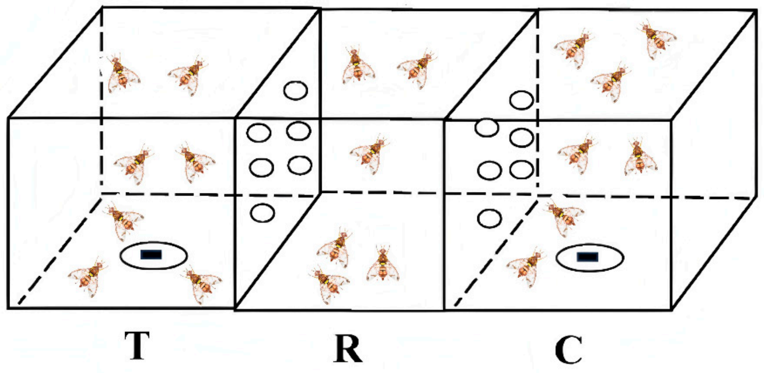

Figure S1. Three-cage olfactometer. C: control cage; T: odorants cage; R: release cage.

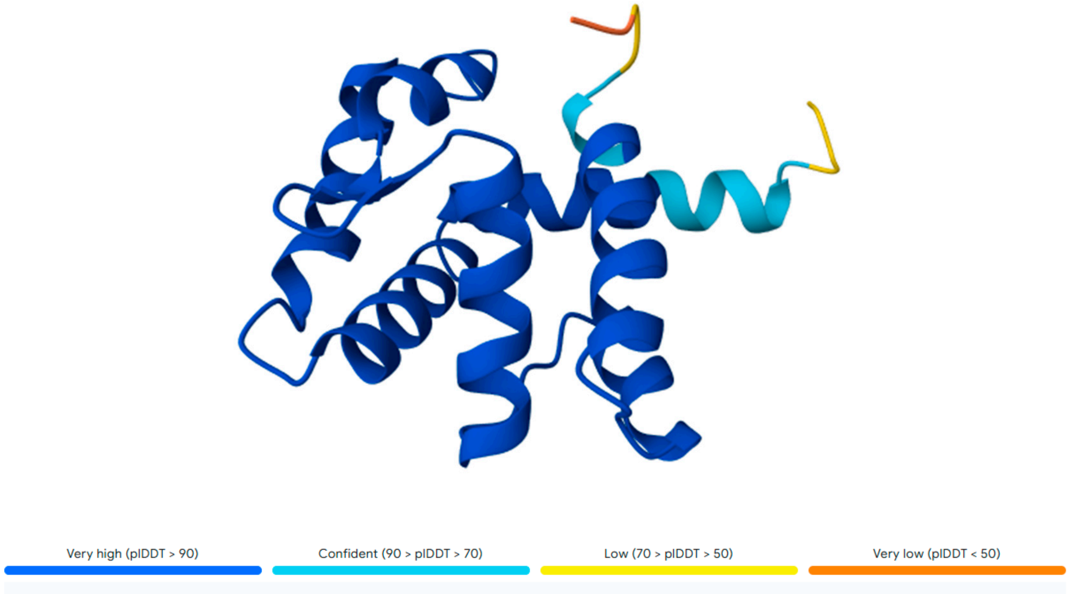

Figure S2. The 3D structure model colored by pLDDT scores of ZcucOBP14 predicted by AlphaFold3.

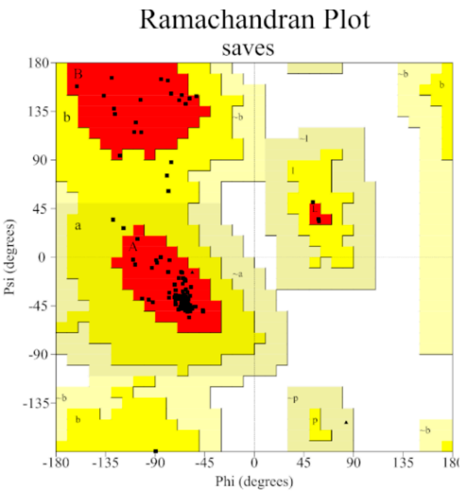

Plot statistics

Residues in most favoured regions [A,B,L]	116	94.3%
Residues in additional allowed regions [a,b,l,p]	7	5.7%
Residues in generously allowed regions [~a,~b,~l,~p]	0	0.0%
Residues in disallowed regions	0	0.0%
	----	------
Number of non-glycine and non-proline residues	123	100.0%
Number of end-residues (excl. Gly and Pro)	1	
Number of glycine residues (shown as triangles)	6	
Number of proline residues	4	

Total number of residues	134	

Overall quality factor**: 100.000

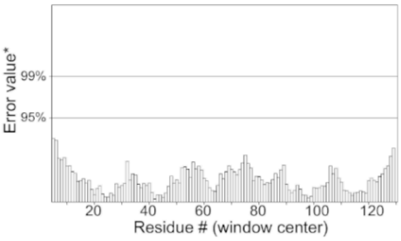

*On the error axis, two lines are drawn to indicate the confidence with which it is possible to reject regions that exceed that error value.

**Expressed as the percentage of the protein for which the calculated error value falls below the 95% rejection limit. Good high resolution structures generally produce values around 95% or higher. For lower resolutions (2.5 to 3A) the average overall quality factor is around 91%.

Figure S3. Model quality evaluation of ZcucOBP14 model
